# P02-04 Citizen science during Covid-19 pandemic to enhance an activating environment in a low-SES neighborhood

**DOI:** 10.1093/eurpub/ckac095.023

**Published:** 2022-08-29

**Authors:** Berry Van Holland, Nikki Jepkema, Johan De Jong

**Affiliations:** School of Sports Studies, Hanze University of Applied Sciences, Groningen, The Netherlands

**Keywords:** citizen science, social innovation, community of practice, active lifestyle

## Abstract

One neighborhood in Groningen, the Netherlands, is a neighborhood housing about 12,000 citizens with on average a low-SES background, showing a less healthy and active lifestyle. In the past, initiatives have been undertaken to promote active lifestyle by implementing outdoor facilities stimulating physical activity. However, use of facilities was poor due to lack of citizen involvement. Aim of this project was to engage citizens in the overall process of capturing, plan making and prototyping of concepts for an exercise-friendly physical and social environment.

From January 2020 - May 2022 a Living Lab was run following the ‘Our Voice’ citizen science method. Participatory citizen science was applied in which a community of stakeholders (public/private parties) and citizens was built. The community addressed the problem by creating more insight in promoting/degrading features in the neighborhood concerning an active lifestyle. Citizens (n = 40) used the Stanford Neighborhood Discovery Tool, which allowed for systematic observations of the physical environment. Additionally, emergent research walks gave extra information on neighborhood barriers/facilitators next to Discovery Tool data. Collected data allowed citizens to brainstorm on possible solutions in sessions facilitated by the researchers. Solutions were presented to local government and further developed for implementation and realization.

Use of the Discovery Tool created an overview of the neighborhood. Based on positive/negative features, new ideas were generated for improving exercise-friendliness. One example was a walking route along art objects in the neighborhood. Furthermore, a citizens work group was formed which discussed this route, and other ideas and prototypes, with local government. This group was also involved in realization of prototypes.

Our project resulted in a citizen science approach which can be transferred to other neighborhoods. Use of Discovery Tool showed many benefits for neighborhood plan making. Early and continuous involvement of citizens will lead to more sustainable engagement and is a powerful method to create engagement around societal problems and social innovation in the field of Health Enhancing Physical Activity.

A transferable method for neighborhood development based on citizen science was developed. Key feature in our method was integration of design thinking, citizen engagement, and use of digital tools.

